# LCN2-mediated ferroptosis resistance in tissue homeostasis and early-stage tumorigenesis of the fallopian tube epithelium

**DOI:** 10.1016/j.isci.2025.112654

**Published:** 2025-05-13

**Authors:** Keiyo Imaeda, Tomohiro Tamura, Shimpei Nagai, Eiji Sugihara, Juntaro Yamasaki, Yuji Otsuki, Kohei Nakamura, Takashi Takeda, Kanako Nakamura, Yuya Nogami, Kosuke Tsuji, Tatsuyuki Chiyoda, Iori Kisu, Yusuke Kobayashi, Kouji Banno, Rui Yamaguchi, Kazuhiro Sakurada, Hideyuki Saya, Daisuke Aoki, Ahmed Ashour Ahmed, Osamu Nagano, Kenta Masuda, Wataru Yamagami

**Affiliations:** 1 Department of Obstetrics and Gynecology, Keio University School of Medicine, Tokyo, Japan; 2 Department of Extended Intelligence for Medicine, The Ishii-Ishibashi Laboratory, Keio University School of Medicine, Tokyo, Japan; 3 Division of Gene Regulation, Oncology Innovation Center, Fujita Health University, Toyoake, Japan; 4 Center for Cancer Genomics, Keio University School of Medicine, Tokyo, Japan; 5 Ovarian Cancer Cell Laboratory, MRC Weatherall Institute of Molecular Medicine, University of Oxford, Oxford OX3 9DS, UK; 6 Nuffield Department of Women’s & Reproductive Health, University of Oxford, Oxford OX3 9DU, UK; 7 Department of Gynaecological Oncology, Churchill Hospital, Oxford University Hospitals, Oxford OX3 7LE, UK; 8 Oxford NIHR Biomedical Research Centre, Oxford OX4 2PG, UK; 9 Division of Cancer Systems Biology, Aichi Cancer Center Research Institute, Nagoya, Japan; 10 Division of Cancer Informatics, Nagoya University Graduate School of Medicine, Nagoya, Japan

**Keywords:** Tissue Engineering, Cell biology, Omics

## Abstract

While the fallopian tube epithelium (FTE) is known to be composed of various differentiated cells such as secretory and ciliated cells, the upstream regulatory mechanisms of cell differentiation that are essential for tissue homeostasis remain under investigation. In this study, we established human FTE organoids and identified quiescent cells within the early organoid formation by observing cellular proliferation heterogeneity. We also analyzed two single-cell transcriptomic data to trace the differentiation trajectory in human FTE, and found that the gene *LCN2* serves as a marker gene of early stage of the trajectory. Genetically manipulated FTE organoids indicated that LCN2 inhibits ferroptosis and promotes cell survival under oxidative stress. In addition, the FTE organoids introduced p53 dysfunction, the common genetic characteristics of high-grade serous carcinoma, showed upregulated *LCN2* expression and enhanced ferroptosis resistance. This study provides insights into the LCN2-mediated protective mechanism of human FTE quiescent cells and its potential role in tumorigenesis.

## Highlights

• Human FTE organoids recapitulate proliferative heterogeneity with quiescent cells
• Quiescent undifferentiated cells are characterized by NF-κB signaling and LCN2
• LCN2 protects FTE cells from ferroptosis and supports cell survival
• p53 dysfunction confers NF-κB activation and LCN2-mediated ferroptosis resistance

## Graphical abstract

**Figure:**
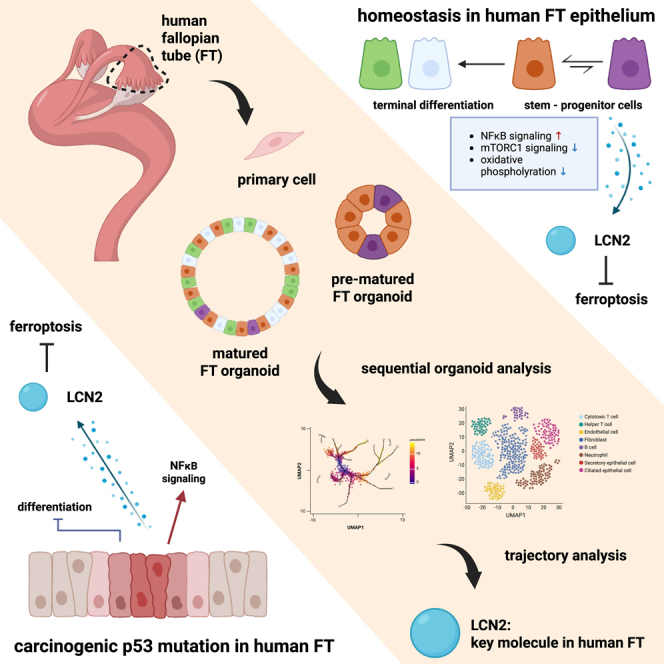

## Introduction

Understanding the normal histological structure of an organ is crucial as it improves our knowledge of the underlying mechanisms governing its function and disease development. The human fallopian tube (FT) is an essential component of the female reproductive system, and a potential site of origin of high-grade serous carcinoma (HGSC), the most common subtype of ovarian cancer.^1^ The human FT is divided into three segments, each with distinct functions: the fimbriae, which captures the oocyte post-ovulation; the ampulla wherein fertilization occurs; and the isthmus, which transports the embryo to the uterus for implantation.^2^ Recent studies have mapped the cellular composition of human FT and revealed that it comprises epithelial, stromal, and immune cells.^3^^,^^4^^,^^5^ The interaction between these various cell types within the human FT is essential for normal functioning.^6^^,^^7^

The FT is a dynamic organ, with its epithelium (FTE) exposed to a rich and diverse microenvironment, regulated by several hormones, growth factors, and ions during each ovulation. For instance, efficient reproduction is regulated by the sex hormones, estrogen, and progesterone which modulate the differentiation of FTE cells.^8^ Estrogen plays a role in the epithelial growth factor receptor (EGFR) pathway, and promotes the terminal differentiation of FTE cells into ciliated cells.^8^^,^^9^ As described, the differentiation of human FTE cells, which is crucial for the proper functioning of the reproductive organ, is regulated by a complex microenvironment.

Although ciliated cells are recognized as the final stage in FTE differentiation, the upstream cells in the differentiation process remain under investigation. Previous studies have shown that the human FT is a rich source of mesenchymal stem cells, and have also been reported to detect quiescent stem-like cells in the mouse FTE.^10^^,^^11^^,^^12^^,^^13^ Additionally, undifferentiated stem-like cells in the human FTE reside in the distal portion of the tube.^7^ In general, quiescent tissue stem cells are characterized by their slow division rate and ability to maintain a dormant state, protecting themselves from the accumulation of genetic mutations under oxidative stress and hormonal changes.^14^^,^^15^ These quiescent cells are crucial for maintaining tissue homeostasis. However, these quiescent cells can undergo oncogenic transformation when exposed to prolonged stress or mutagenic agents, potentially leading to cancer.^16^ Investigating the protective mechanisms of these crucial quiescent cells against various environmental factors, such as oxidative stress, is essential for understanding tissue abnormalities, such as tumorigenesis.

A recent study revealed that organoids established from the human FT can recapitulate cellular differentiation and are expected to help elucidate the stemness of the human FTE.^17^ Therefore, in this study, we first aimed to establish human FTE organoids and identify quiescent cells within the early organoid formation through the analysis of cellular proliferation heterogeneity.

## Results

### Establishment and characterization of human FTE organoids

**Figure 1:**
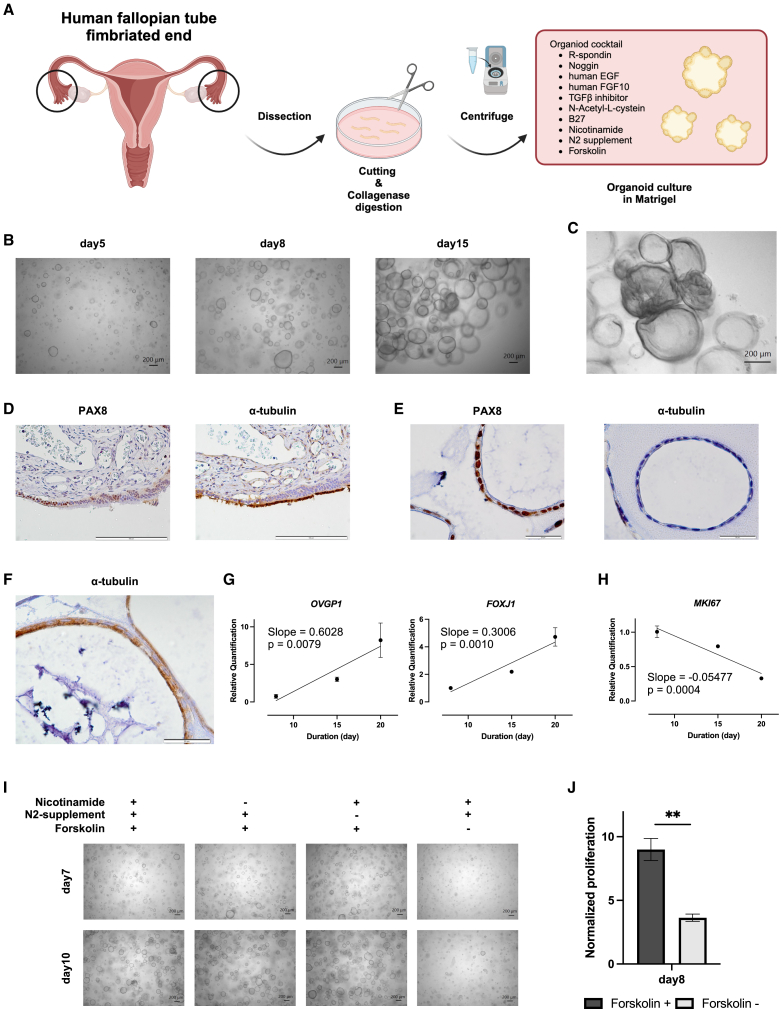
Establishment and characterization of human FTE organoids (A) Schematic representation of the process of establishing human FTE organoids. Created with BioRender.com. (B) Bright-field images of the human FTE organoids on days 5, 8, and 15. Scale bars indicate 200 μm. (C) A bright-field image of the human FTE organoids on day 17. A scale bar indicates 200 μm. (D) Immunohistochemistry of a human FT stained with an anti-PAX8 antibody and an anti-α-tubulin antibody. Scale bars indicate 100 μm. (E) Immunohistochemistry of the human FTE organoids on day 15 stained with an anti-PAX8 antibody and an anti-α-tubulin antibody. Scale bars indicate 20 μm. (F) Immunohistochemistry of the human FTE organoids on day 20 stained with an anti-α-tubulin antibody. A scale bar indicates 20 μm. (G) Expression of the secretory cell marker gene *OVGP1* and the ciliated cell marker gene *FOXJ1* examined using qPCR. The results on days 8, 15, and 20 are shown (*n* = 2, with three technical replicates per sample). (H) Expression of the proliferation marker gene *MKI67* examined using qPCR. The results on days 8, 15, and 20 are shown (*n* = 2, with three technical replicates per sample). (I) Bright-field images of the human FTE organoids cultured in the organoid culture medium and the organoid culture medium lacking nicotinamide, N2 supplement, or forskolin, on days 7 and 10. Scale bars indicate 200 μm. (J) Growth level of the FTE organoids cultured in the organoid culture medium or the organoid culture medium lacking forskolin on day 8, normalized to their growth level on day 3 (*n* = 2, with three technical replicates per sample). Data are represented as the mean ± SEM, in principle. Statistical analyses were performed using the extra sum-of-squares F test for linear regression in Figures 1G and 1H, and the Student’s *t* test in Figure 1J. Significance levels are ∗*p* < 0.05, ∗∗*p* < 0.01, ∗∗∗*p* < 0.001, and ∗∗∗∗*p* < 0.0001.

To recapitulate and analyze the nature of the human FTE *in vitro*, we established human FTE organoids from a human FT clinical sample (Figure 1A). Organoid culture is a three-dimensional culture method of epithelial cells, and has facilitated culturing and passaging of epithelial cells *in vitro*. The following factors have been reported to be necessary for human FTE organoid culture, 4% R-spondin, 2% Noggin, 100 ng/mL human EGF, 100 ng/mL human FGF10, 5 μM A83-01, 1 mM N-acetylcysteine, 2% B27, 1 mM nicotinamide, 1% N2 supplement, and 10 μM forskolin.^6^ We digested the human FT clinical sample with collagenase and embedded the digested cells in Matrigel; consequently, the human FTE organoids were successfully established by adding an organoid culture cocktail containing the necessary factors (R-spondin, Noggin, human EGF, human FGF10, A83-01, N-acetylcysteine, B27, nicotinamide, N2 supplement, forskolin) (Figure 1B). Upon observing the organoids sequentially, it was found that the folded structure gradually emerged from day 15 (Figure 1C). Immunohistochemistry (IHC) of the human FT showed that the tube epithelium comprised PAX8-positive secretory cells and α-tubulin–positive ciliated cells (Figure 1D). IHC of the human FTE organoids showed that PAX8 was well-stained, but α-tubulin was not stained on day 15 (Figure 1E). α-tubulin was found to be well-stained on the cellular surface on day 20 (Figure 1F), which indicated that the human FTE organoids recapitulated the histology of human FT. Additionally, the ciliary differentiation in the folded structures took over 15 days, which is consistent with a previous report.^18^

Next, we examined the changes in gene expression relevant to human FTE differentiation and proliferation through long-term culture (days 8, 15, and 20) using quantitative PCR (qPCR). Although the expression of the secretory cell marker *OVGP1* and ciliated cell marker *FOXJ1* increased from days 8–20 (Figure 1G), the expression of the proliferation marker *MKI67* decreased from days 8–20 (Figure 1H). This suggests that as the human FTE organoids differentiated, the proliferation of the organoids gradually downregulated.

To explore the important factors comprising the organoid culture cocktail for primary organoid formation, the primary proliferation of the human FTE organoids cultured in the organoid culture medium lacking nicotinamide, N2 supplement, or forskolin was examined. While the primary proliferation of the organoids was not inhibited in the culture medium lacking nicotinamide or N2 supplement, the primary proliferation of the organoids was inhibited in the culture medium lacking forskolin (Figures 1I and 1J), which indicated that forskolin-induced cAMP synthesis from ATP is essential for the primary formation of human FTE organoids.

From these results, it was found that individual cells with the ability to form an organoid expand rapidly using cAMP and maintain themselves undifferentiated in a week (early-culture organoids); conversely, these pre-matured cells in the early-culture FTE organoids undergo cellular differentiation and cell-cycle arrest in the next weeks.

### Quiescent cells exist in early-culture organoids and exhibit different transcriptomic characteristics

Stemness in human tissues has been discussed mainly from two perspectives: the cellular biological characteristics such as self-renewal, pluripotency, and dormancy and the expression of key genes such as *LGR5* and *PROM1*.^18^^,^^19^ Although the stemness of human FTE has been studied for decades, no definitive key gene associated with stemness has been identified. This makes it challenging to define stemness in human FTE based solely on gene expression, and we must focus on the cellular characteristics of stem cells to identify them among the numerous epithelial cells in the human FTE. It has been reported that quiescent cells exist in the mouse FTE.^12^^,^^13^ We first investigated whether the proliferation heterogeneity exists in the human FTE organoids *in vitro*.

**Figure 2:**
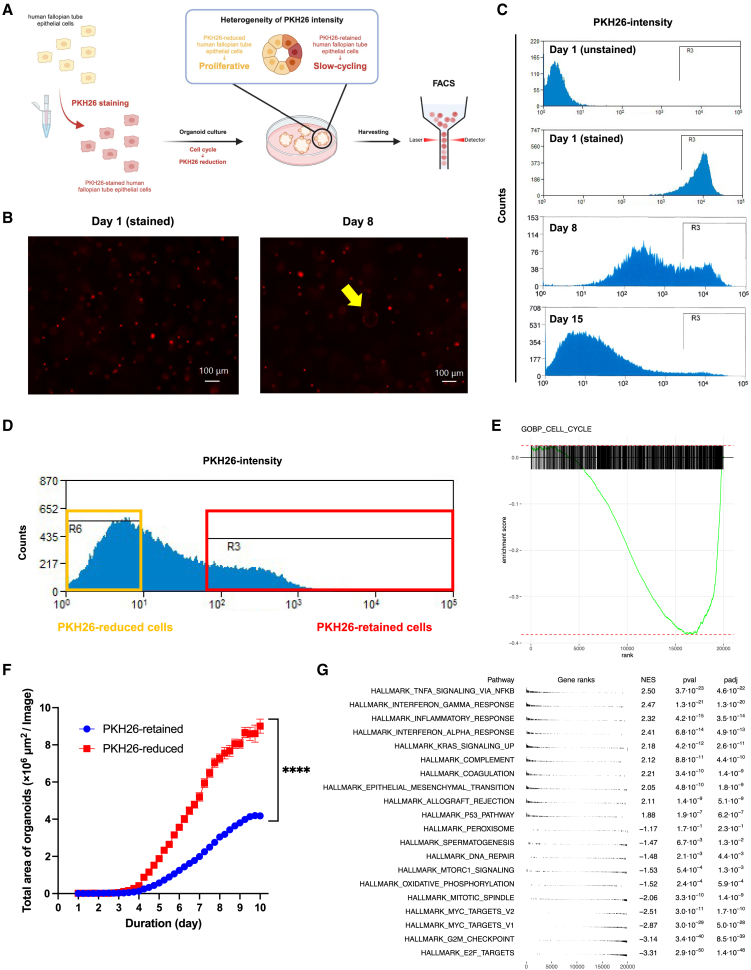
Quiescent cells in the early-culture FTE organoids (A) Schematic representation of cell sorting based on proliferation using PKH26. PKH26 is a red fluorescent dye that gradually decreases upon cell division. Created with BioRender.com. (B) Fluorescence images of the human FTE organoids stained with PKH26 on day 1 after staining and on day 8. Scale bars indicate 100 μm. (C) Fluorescence intensity of PKH26 on individual cells consisting of human FTE organoids stained with PKH26 on day 1 before staining, day 1 after staining, and days 8 and 15. (D) Fluorescence intensity standard used to sort individual cells consisting of the human FTE organoids stained with PKH26 into PKH26-reduced, proliferative cells and PKH26-retained, quiescent cells. (E) A bar cord plot of a cell cycle gene set in gene ontology biological processes. Genes from the bRNA-seq (the PKH26-retained cells vs. the PKH26-reduced cells) were ranked by the normalized ratio of TPM values of the PKH26-retained cells to those of the PKH26-reduced cells. (F) Total area of organoids from the PKH26-retained cells or the PKH26-reduced cells (*n* = 1, with eight technical replicates per samples). (G) A list of the top 10 and bottom 10 hallmark gene sets enriched in the PKH26-retained cells (bottom: enriched in the PKH26-reduced cells) ranked by *p*-values. Genes from the bRNA-seq (the PKH26-retained cells vs. the PKH26-reduced cells) were ranked by the normalized ratio of TPM values of the PKH26-retained cells to those of the PKH26-reduced cells. Gene ranks, NES, and *p*-values are shown. Data are represented as the mean ± SEM, in principle. Statistical analysis was performed using the Student’s *t* test in Figure 2F. Significance levels are ∗*p* < 0.05, ∗∗*p* < 0.01, ∗∗∗*p* < 0.001, and ∗∗∗∗*p* < 0.0001.

To evaluate the growth rate of individual cells in the human FTE organoids, FTE cells were stained with a red fluorescent membrane dye PKH26 (day 1), and after culturing, the fluorescence intensity of individual cells comprising the human FTE organoids was measured using FACS (Figure 2A). PKH26 is a dye anchored to the cellular membrane, and is thought to decrease upon cell division; therefore, we can define PKH26-retained cells as quiescent cells and PKH26-reduced cells as proliferative cells. Since the gradation of PKH26 fluorescence in the human FTE organoids was observed following organoid culture, heterogeneous proliferation was demonstrated in the human FTE organoids (Figure 2B). Next, we sequentially evaluated the reduction rate of PKH26 fluorescence intensity using FACS. As a result, two distinct cell populations with different reduction rates of PKH26 fluorescence intensity were observed after a week (Figure 2C). Though the qPCR results using the whole RNA on days 8, 15, and 20 showed a proliferative and undifferentiated property of the early-culture organoids (Figure 1H), it was found that some cells remain quiescent during an organoid expansion in the first week. Thus, we sorted individual stained cells in the human FTE organoids into PKH26-retained cells (whose fluorescence intensity was at the level almost equal to the intensity of primary stained-cells at day 1) and PKH26-reduced cells (whose fluorescence intensity was almost at the same level as a negative control) by FACS on day 6 (Figure 2D).

Subsequently, to validate the proliferative characteristics of the PKH26-reduced cells compared with the PKH26-retained cells, the gene expression patterns were analyzed by bulk RNA sequencing (bRNA-seq). Gene set enrichment analysis (GSEA) in terms of a cell cycle gene set in the gene ontology biological process showed that the expression of genes related to cell cycle was downregulated in the PKH26-retained cells (Figure 2E). In addition, we performed an organoid formation assay and compared the rate of organoid area expansion between the PKH26-reduced cells and PKH26-retained cells. As a result, the expansion rate of the PKH26-reduced cells was higher than that of the PKH26-retained cells (Figure 2F). These results suggest that individual cells in the early-culture FTE organoids were successfully classified based on their proliferation rate into quiescent cells and proliferative cells.

Next, we compared the gene expression patterns between the PKH26-retained cells and PKH26-reduced cells by GSEA in terms of hallmark gene sets. As a result, it was found that both mTORC1 signaling and oxidative phosphorylation in addition to cell cycle-related gene sets were downregulated in the PKH26-retained cells (Figure 2G). These transcriptomic features are similar with the characteristics of quiescent stem cells, previously reported in other tissues.^14^^,^^20^ Interestingly, inflammatory reactions relevant to NF-κB signaling were upregulated in the PKH26-retained cells. NF-κB signaling canonically functions in immune cells as an inflammatory mediator; however, the function of NF-κB signaling in epithelial cells has not been well studied.

### Gradation of the stem-like cell properties in human FTE single-cell transcriptome data

Though the transcriptomic analysis of PKH26-retained cells and PKH26-reduced cells showed the difference between quiescent cells and proliferative cells in the human early-culture FTE organoids, this analysis captured only the early part of cellular differentiation. The human FTE contains other types of epithelial cells, and it is necessary to investigate all the cell types in human FTE and to understand a distribution of the quiescent cell properties in the differentiation trajectory.

**Figure 3:**
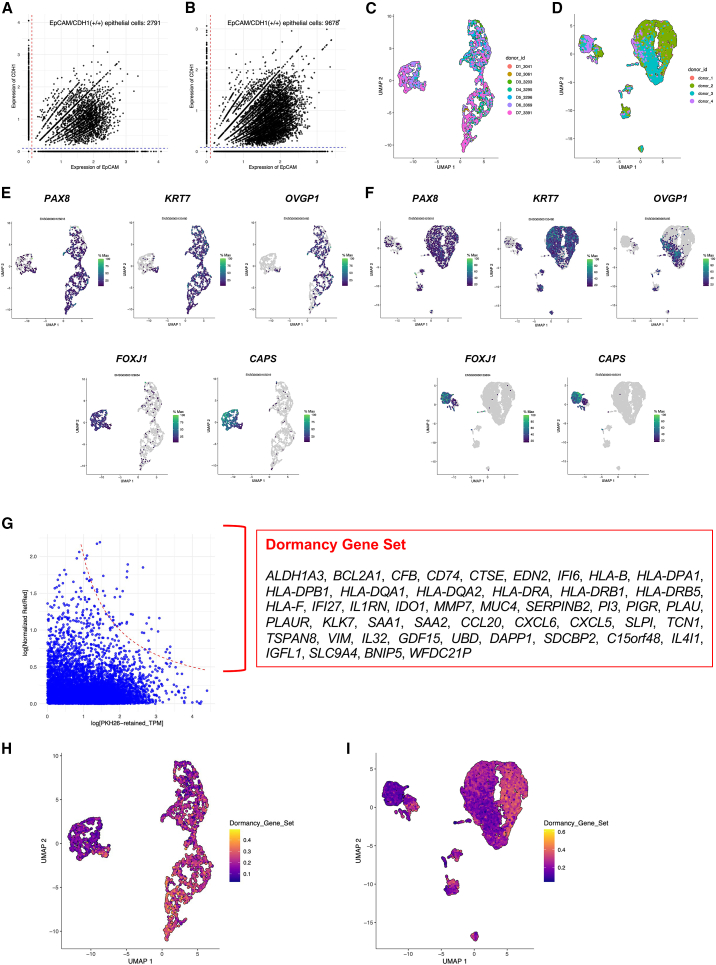
Gradation of the stem-like cell characteristics and the NF-κB signaling activation in two independent human FT single-cell RNA sequencing data (A) A dot plot representing the normalized expression of *EpCAM* (the x axis, Ensembl ID: ENSG00000119888) and *CDH1* (the y axis, Ensembl ID: ENSG00000039068) in the scRNA-seq data by Lengyel et al. Total counts of *EpCAM*/*CDH1* (+/+) cells are 2,791. The red and blue lines were “x = 0.1” and “y = 0.1” respectively. (B) A dot plot representing the normalized expression of *EpCAM* (the x axis, Ensembl ID: ENSG00000119888) and *CDH1* (the y axis, Ensembl ID: ENSG00000039068) in the scRNA-seq data by Ulrich et al. Total counts of *EpCAM*/*CDH1* (+/+) cells are 9,678. The red and blue lines were “x = 0.1” and “y = 0.1” respectively. (C) Batch effect-removed UMAP of the epithelial cells in the scRNA-seq data by Lengyel et al. Each cell is annotated by the donor ID of the sample. (D) Batch effect-removed UMAP of the epithelial cells in the scRNA-seq data by Ulrich et al. Each cell is annotated by the donor ID of the sample. (E) The UMAP with the expression values of ubiquitous secretory cell marker genes *PAX8* (Ensembl ID: ENSG00000125618), *KRT7* (Ensembl ID: ENSG00000135480); a late-stage secretory cell marker gene *OVGP1* (Ensembl ID: ENSG00000085465); ciliated cell marker genes *FOXJ1* (Ensembl ID: ENSG00000129654) and *CAPS* (Ensembl ID: ENSG00000105519) in the scRNA-seq data by Lengyel et al. The expression values are normalized by the R package “monocle3”. (F) The UMAP with the expression values of ubiquitous secretory cell marker genes *PAX8* (Ensembl ID: ENSG00000125618), *KRT7* (Ensembl ID: ENSG00000135480); a late-stage secretory cell marker gene *OVGP1* (Ensembl ID: ENSG00000085465); ciliated cell marker genes *FOXJ1* (Ensembl ID: ENSG00000129654) and *CAPS* (Ensembl ID: ENSG00000105519) in the scRNA-seq data by Ulrich et al. The expression values are normalized by the R package “monocle3”. (G) A dot plot of the bRNA-seq result (the PKH26-retained cells vs. the PKH26-reduced cells); the x axis indicates the log-scale TPM values of the PKH26-retained cells, and the y axis indicates the log-scale normalized ratio of TPM values of the PKH26-retained cells to TPM values of the PKH26-reduced cells. The formula of the red curve is as follows: (the log-scale TPM values of the PKH26-retained cells) × (the log-scale normalized ratio of TPM values of the PKH26-retained cells to TPM values of the PKH26-reduced cells) = 2. We defined the 47 genes located above the red curve as the dormancy gene set. (H) The UMAP of the scRNA-seq data by Lengyel et al., indicating the enrichment score of the dormancy gene set calculated by the R package “escape” and its dependency “AUCell”. (I) The UMAP of the scRNA-seq data by Ulrich et al., indicating the enrichment score of the dormancy gene set calculated by the R package “escape” and its dependency “AUCell”.

To investigate the intra-tissue heterogeneity of human FTE, we analyzed two independent scRNA-seq published data of the human FT reported by Lengyel et al. and Ulrich et al.^4^^,^^5^ First, to create epithelial cell subsets from the scRNA-seq data by Lengyel et al. (total cell counts: 60,574 cells) and by Ulrich et al. (total cell counts: 59,738 cells) respectively, epithelial cell marker genes *EpCAM/CDH1* (+/+) cells were extracted based on normalized expression values of these genes (Lengyel et al.: 2,971 cells, Ulrich et al.: 9,678 cells) (Figures 3A and 3B). Next, to investigate the biologically significant variety of cellular characteristics in the human FTE, a dimensional reduction was conducted and unfavorable batch effects, due to the difference in donors of the FT samples, were removed (Figures 3C and 3D). By evaluating the expression of conventional differentiation markers—*PAX8*, *KRT7* (secretory cell marker genes); *OVGP1* (a late-stage secretory cell marker gene); and *FOXJ1* and *CAPS* (ciliated cell marker genes)—on the uniform manifold approximation and projection (UMAP), it was confirmed that the expression of the secretory cell marker genes and that of the ciliated cell marker genes were exclusive, and the expression of *OVGP1* varied among *KRT7*/*PAX8* (+/+) secretory cells (Figures 3E and 3F). These findings indicate the presence of a gradation in differentiation status within the human FTE.

To locate the PKH26-retained, quiescent cells in the human FTE organoids on the UMAP, the bRNA-seq expression data was searched for upregulated genes in the PKH26-retained cells using the following formula: log_10_(normalized TPM ratio of the PKH26-retained cells to the PKH26-reduced cells) × log_10_(TPM value of the PKH26-retained cells) > 2, and a dormancy gene set including 47 genes (Figure 3G) was obtained. Subsequently, we performed AUCell, an enrichment analysis optimized for scRNA-seq data, to evaluate the dormancy gene set in two scRNA-seq datasets (the scRNA-seq dataset by Lengyel et al. included 47/47 genes in the dormancy gene set, and the dataset by Ulrich et al. included 46/47 genes, excluding *WFDC21P*). As a result, it was found that the PKH26-retained, quiescent cells with an upregulated dormancy gene set were located within the *OVGP1*^*LOW*^ secretory cell regions (Figures 3H and 3I).

### Trajectories of cellular differentiation from the PKH26-retained, quiescent undifferentiated cells in human FTE

**Figure 4:**
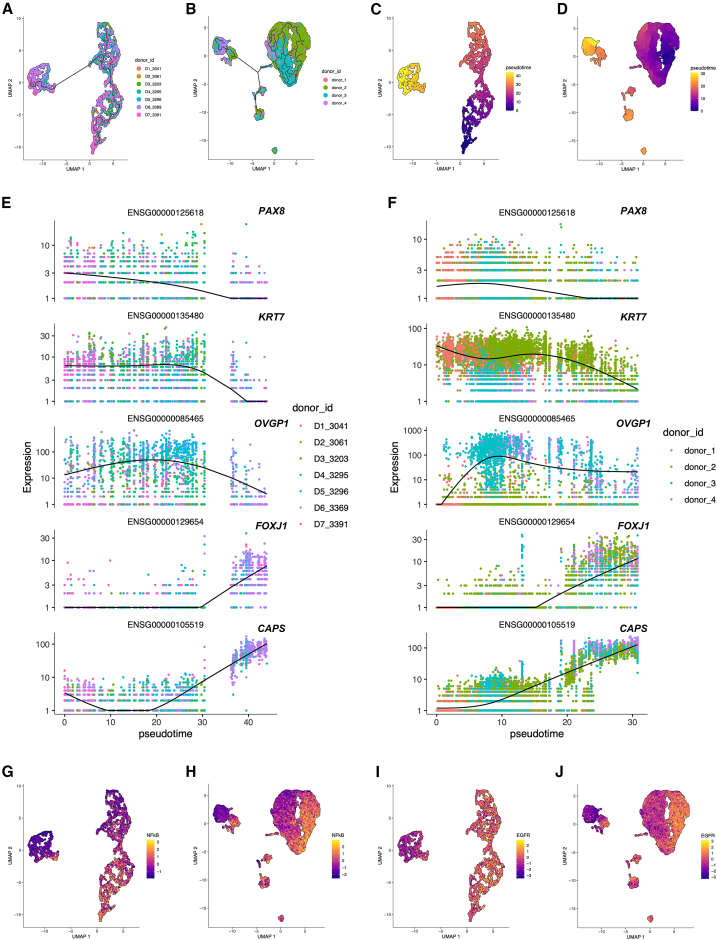
Trajectory of epithelial cell differentiation in human FTE and pathway activation analysis (A) The UMAP of the scRNA-seq data by Lengyel et al. and the trajectory described by the R package “monocle3”. Each cell is annotated by the donor ID of the sample. (B) The UMAP of the scRNA-seq data by Ulrich et al. and the trajectory described by the R package “monocle3”. Each cell is annotated by the donor ID of the sample. (C) The UMAP of the scRNA-seq data by Lengyel et al., indicating the pseudotime calculated by the R package “monocle3”. The node nearest to the cells with the highest enrichment score of the dormancy gene set is defined as the root node of the trajectory. (D) The UMAP of the scRNA-seq data by Ulrich et al., indicating the pseudotime calculated by the R package “monocle3”. The node nearest to the cells with the highest enrichment score of the dormancy gene set is defined as the root node of the trajectory. (E) The transition of the expression of the genes *PAX8* (Ensembl ID: ENSG00000125618), *KRT7* (Ensembl ID: ENSG00000135480), *OVGP1* (Ensembl ID: ENSG00000085465), *FOXJ1* (Ensembl ID: ENSG00000129654), and *CAPS* (Ensembl ID: ENSG00000105519) along the pseudotime of the scRNA-seq data by Lengyel et al. modeled by the R package “monocle3”. The black curves indicate the transition, and the colored dots indicate the expression value of each cell. (F) The transition of the expression of the genes *PAX8* (Ensembl ID: ENSG00000125618), *KRT7* (Ensembl ID: ENSG00000135480), *OVGP1* (Ensembl ID: ENSG00000085465), *FOXJ1* (Ensembl ID: ENSG00000129654), and *CAPS* (Ensembl ID: ENSG00000105519) along the pseudotime of the scRNA-seq data by Ulrich et al. modeled by the R package “monocle3”. The black curves indicate the transition, and the colored dots indicate the expression value of each cell. (G) The UMAP of the scRNA-seq data by Lengyel et al., indicating the pathway activation score of NF-κB signaling calculated by the R package “progeny”. (H) The UMAP of the scRNA-seq data by Ulrich et al., indicating the pathway activation score of NF-κB signaling calculated by the R package “progeny”. (I) The UMAP of the scRNA-seq data by Lengyel et al., indicating the pathway activation score of EGFR signaling calculated by the R package “progeny”. (J) The UMAP of the scRNA-seq data by Ulrich et al., indicating the pathway activation score of EGFR signaling calculated by the R package “progeny”.

The enrichment analysis of the dormancy gene set revealed the intra-tissue heterogeneity of transcriptomic status in the human FTE. The next aim involved understanding the sequence of the transcriptomic transition from the PKH26-retained, quiescent cells in the human FTE. Firstly, a trajectory of the transcriptomic transition was constructed on the UMAP (Figures 4A and 4B). Next, a node closest to the FTE cells with the highest enrichment score of the dormancy gene set as an origin of the trajectory was defined, and pseudotime was calculated from the root node (Figures 4C and 4D).

To examine the assumption that the trajectories reflect the process of human FTE differentiation, the transition of the expression of the conventional human FTE differentiation marker genes was analyzed (*PAX8*, *KRT7*, *OVGP1*, *FOXJ1*, and *CAPS*) along the pseudotime. As a result, the expression of *OVGP1* had a peak at the middle part of the pseudotime, and the expression of *FOXJ1* and *CAPS* increased exponentially at the last part of the pseudotime (Figures 4E and 4F). *PAX8* and *KRT7* were highly expressed at the early and middle parts of the pseudotime. These findings indicated that the pseudotime based on the dormancy gene set scores reflected the differentiation process of the human FTE, and enabled the analysis of the very early stages of the differentiation.

Additionally, we conducted a pathway analysis to investigate the activation of several pathways, especially NF-κB signaling that was particularly activated in the PKH26-retained cells. As a result, NF-κB signaling was activated at the early part of the trajectories, and the activation decreased with pseudotime in the secretory cell region (Figures 4G and 4H). Interestingly, we also found that EGFR signaling was downregulated in the ciliated cell region (Figures 4I and 4J). EGFR signaling has been reported as a regulator of ciliogenesis.^8^^,^^21^ From these pathway analysis results, it was found that several pathways, such as NF-κB signaling, play a role in the cellular differentiation within the human FTE.

### *In silico* scRNA-seq exploration detects candidates of stem cell marker genes in human FTE

**Figure 5:**
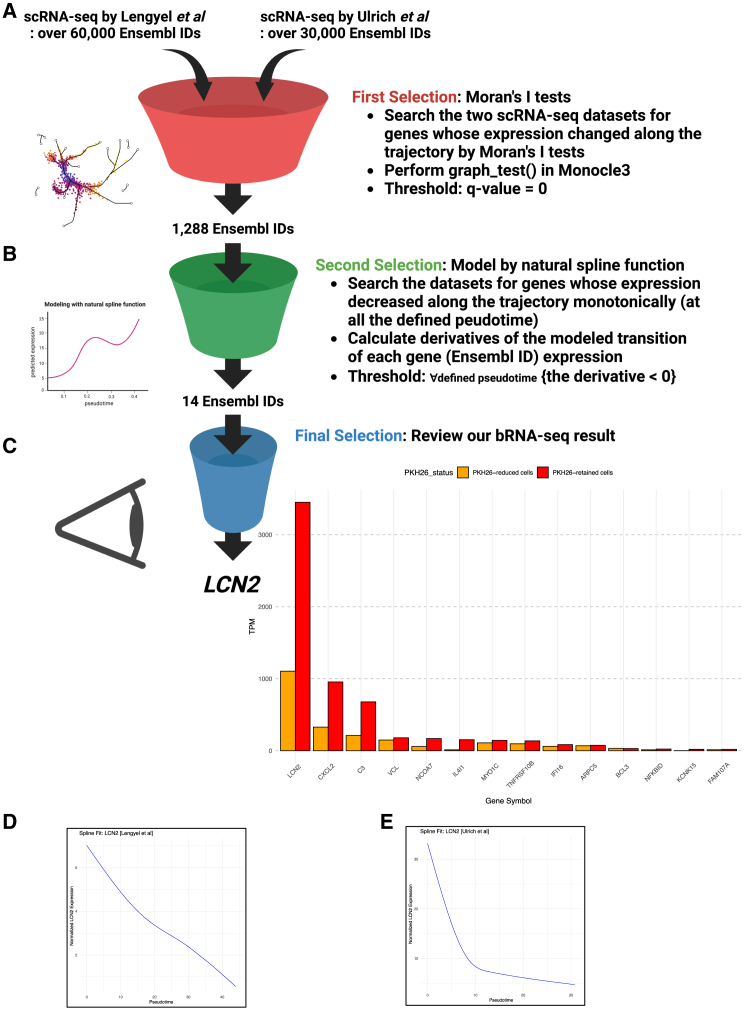
*In silico* exploration for stem cell marker genes of human FTE (A) Schematic representation of the first selection of the key genes in the stemness of human FTE. (B) Schematic representation of the second selection of the key genes in the stemness of human FTE. (C) Schematic representation of the final selection of the key genes in the stemness of human FTE, and bar charts indicating TPM values of the final 14 genes in the bRNA-seq result. (D) The transition of the expression of LCN2 (Ensembl ID: ENSG00000148346) in the scRNA-seq data by Lengyel et al. modeled by the R package "splines2”. (E) The transition of the expression of LCN2 (Ensembl ID: ENSG00000148346) in the scRNA-seq data by Ulrich et al. modeled by the R package “splines2”.

The bRNA-seq data of the PKH26-retained, quiescent cells and the PKH26-reduced, proliferative cells revealed 47 significant genes (the dormancy gene set), which were helpful for building the trajectories on the UMAP; however, the analysis of the human FTE organoids at one time point was not adequate to precisely understand the transcriptomic transition. To detect key genes whose expression varied along the trajectories, the spatial correlation of each gene expression on the UMAP was analyzed using Moran’s I tests. As a result, we obtained 1,363 Ensembl IDs from the scRNA-seq data by Lengyel et al. and 4,157 Ensembl IDs from the data by Ulrich et al. as the genes whose expression changed along the trajectories (Figure 5A). Subsequently, upon extracting the intersection of these genes, 1,288 Ensembl IDs were obtained as important genes for the cellular differentiation in the human FTE.

Next, to analyze the transition of gene expression along the pseudotime, we modeled the transition with natural spline functions and calculated the derivative at each defined pseudotime (Figure 5B). The derivatives reflected whether the gene expression increased or decreased at the defined pseudotime; therefore, we extracted 14 Ensembl IDs with the derivatives that were less than 0 at all the defined pseudotimes, which meant that the gene expression monotonically decreased from the root node at all the defined pseudotimes. The 14 Ensembl IDs represented the following genes: *LCN2*, *CXCL2*, *C3*, *VCL*, *NCOA7*, *IL4I1*, *MYO1C*, *TNFRSF10B*, *IFI16*, *ARPC5*, *BCL3*, *NFKBID*, *KCNK15*, and *FAM107A* (Figure 5C). Reviewing the bRNA-seq result, *LCN2* encoding lipocalin-2 showed the highest TPM values in the PKH26-retained cells, and was expressed in the PKH26-retained cells more than three times as much as in the PKH26-reduced cells. As a gene with the very high expression in the quiescent cells and the middle expression in the proliferative cells during the early organoid formation, we focused on *LCN2*. Finally, we examined the validity of the sequential analysis and found that the modeling with natural spline functions performed well (Figures 5D and 5E).

### LCN2 inhibits ferroptosis by modulating iron ion flux in the human FTE organoids

**Figure 6:**
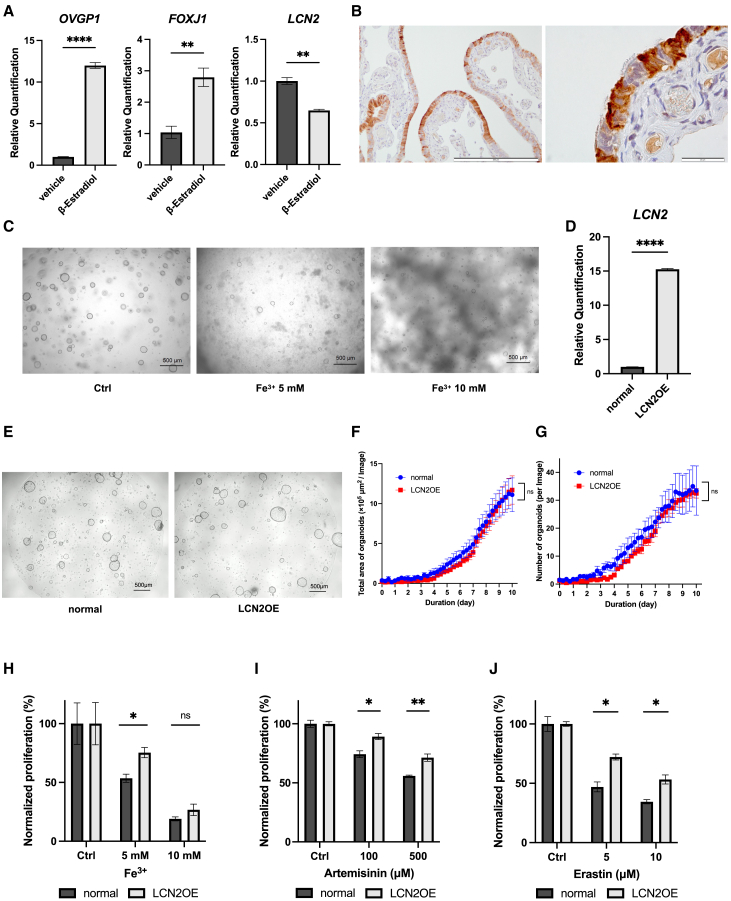
LCN2 inhibits ferroptosis in the human FTE organoids (A) Expression of *OVGP1*, *FOXJ1*, and *LCN2* under treatment with a vehicle or approximately 100 nM β-Estradiol on day 8 examined using qPCR (*n* = 2, with three technical replicates per sample). (B) Immunohistochemistry of a human FT stained with an anti-LCN2-antibody. Images are shown at both low magnification (scale bar: 200 μm) and high magnification (scale bar: 20 μm). (C) Bright-field images of the human FTE organoids cultured under treatment with ferric ions (5 and 10 mM) or a vehicle for 7 days. (D) Expression of *LCN2* in the human FTE organoids and the LCN2-OE organoids examined using qPCR (*n* = 2, with three technical replicates per sample). (E) A Bright-field image of the human FTE organoids and the LCN2-OE organoids on day 10. A scale bar indicates 500 μm. (F) Total area of organoids from the human FTE cells or the LCN2-OE cells (*n* = 1, with four technical replicates per samples). (G) Number of organoids from the human FTE cells or the LCN2-OE cells (*n* = 1, with four technical replicates per samples). (H) Cell viability of the human FTE organoids and the LCN2-OE organoids cultured under treatment with ferric ions (5 and 10 mM) or a vehicle on day 8, normalized to the cell viability under treatment with a vehicle (*n* = 1, with three technical replicates per sample). (I) Cell viability of the human FTE organoids and the LCN2-OE organoids treated with 100 and 500 μM artemisinin, or a vehicle, normalized to the cell viability under treatment with a vehicle (*n* = 1, with three technical replicates per sample). (J) Cell viability of the human FTE organoids and the LCN2-OE organoids treated with 5 and 10 μM erastin, or a vehicle, normalized to the cell viability under treatment with a vehicle (*n* = 1, with three technical replicates per sample). Data are represented as the mean ± SEM, in principle. Statistical analyses were performed using the Student’s *t* tests for all comparisons. Significance levels are ∗*p* < 0.05, ∗∗*p* < 0.01, ∗∗∗*p* < 0.001, and ∗∗∗∗*p* < 0.0001.

To examine the expression of *LCN2* under the cellular differentiation *in vitro*, cellular differentiation of the human FTE organoids was induced by β-estradiol and the expression of *LCN2* was compared. As a result, β-estradiol increased the expression of *OVGP1* and *FOXJ1,* and decreased the expression of *LCN2* to 0.647-fold (Figure 6A). Additionally, IHC of a human FTE sample also revealed the heterogeneous expression of LCN2 in non-ciliated cells (Figure 6B).

The protein LCN2 is associated with immune reactions via ferric ion (Fe^3+^) transportation, and a recent study has reported that the expression of LCN2 was relevant to the activation of NF-κB in cancer cells.^22^^,^^23^ A ferric ion can be reduced to a ferrous ion (Fe^2+^), which then participate in the Fenton reaction to generate reactive oxygen species that can induce ferroptosis, a type of cell death. Recent studies have highlighted the growing interest in the relationship between ferroptosis and gynecologic cancers and reproductive diseases.^24^^,^^25^ To examine the toxic effects of ferric ions on the human FTE, the human FTE organoids were exposed to culture medium containing ferric ions (Figure 6C). As a result, the human FTE organoids underwent cell death in the ferric-ion-containing culture medium, indicating that ferric ions exert a cytotoxic effect against the human FTE.

To investigate the function of LCN2 in the human FTE in terms of ferroptosis, we established human FTE organoids with overexpressed *LCN2* (LCN2-OE) (Figure 6D). The overexpression of *LCN2* did not cause any changes in organoid morphology and organoid formation (Figures 6E–6G); therefore, *LCN2* can be the stem cell marker gene but does not influence an organoid forming ability directly. Next, we performed a toxicity assay using ferric ion-containing culture medium (with ferric ion concentrations of 5 and 10 μM) on both the human FTE organoids and the LCN2-OE organoids. As a result, the LCN2-OE organoids showed resistance to ferric ion-induced ferroptosis (Figure 6H). In addition, to examine the tolerance of human FTE organoids and LCN2-OE organoids to pharmacologically induced ferroptosis, chemosensitivity assays were conducted with artemisinin and Erastin (both ferroptosis inducers); as a result, the LCN2-OE organoids were more tolerant to artemisinin- or erastin-induced ferroptosis (Artemisinin: 1.20-fold at 100 μM, 1.24-fold at 500 μM; erastin: 1.54-fold at 5 μM, 1.54-fold at 10 μM) (Figures 6I and 6J). These results indicated that LCN2 reduces ferroptosis and enables quiescent cells of the human FTE to survive exposure to ferric ions.

### Carcinogenic p53 dysfunction in human FTE suppresses differentiation and enhances ferroptosis resistance

**Figure 7:**
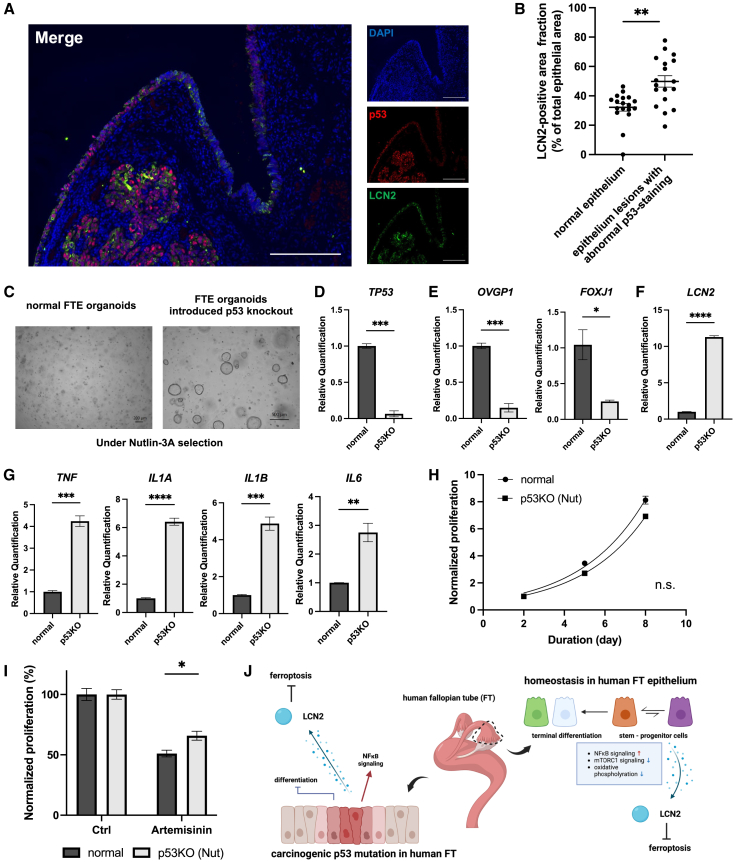
Carcinogenic p53 dysfunction in human FTE suppresses differentiation and enhances ferroptosis resistance (A) Representative fluorescence immunostaining images of very early-stage high-grade serous carcinoma (HGSC, Stage IA). Sections were stained with DAPI (blue), p53 (red), and LCN2 (green), and a merged image to illustrate co-localization. Scale bars indicate ∼250 μm. (B) LCN2-positive area fractions in the IHC of normal epithelium and epithelial lesions with abnormal p53-staining within 18 human FTE samples (including the HGSC Stage IA sample). The LCN2-positive area was quantified, and the percentage of LCN2-positive area in the total epithelial area of each region was calculated using StrataQuest. The percentages of LCN2-positive area are shown. (C) Bright-field images of the human FTE organoids and the human FTE organoids introduced p53 knockout by CRISPR/Cas9 under Nutilin-3A selection. Scale bars indicate 200 or 500 μm. (D) Expression of *TP53* in the human FTE organoids and the p53-knocked single-picked FTE organoids on day 11 examined using qPCR (*n* = 2, with three technical replicates per sample). (E) Expression of *OVGP1* and *FOXJ1* in the human FTE organoids and the p53-knocked single-picked FTE organoids on day 11 examined using qPCR (*n* = 2, with three technical replicates per sample). (F) Expression of *LCN2* in the human FTE organoids and the p53-knocked single-picked FTE organoids on day 11 examined using qPCR (*n* = 2, with three technical replicates per sample). (G) Expression of NF-κB-related genes *TNF*, *IL1A*, *IL1B*, and *IL6* in the human FTE organoids and the p53-knocked single-picked FTE organoids on day 11 examined by qPCR (*n* = 1, with three technical replicates per sample). (H) Growth curve of the human FTE organoids and the p53-knocked FTE organoids selected by Nutlin-3A, normalized to their growth level on day 2 (*n* = 1, with three technical replicates per sample). (I) Proliferation of the human FTE organoids and the p53-knocked FTE organoids selected by Nutlin-3A treated with a vehicle or 500 μM Artemisinin. Data were normalized to those of the vehicle control (*n* = 1, with three technical replicates per sample). (J) Schematic representation of the ferroptosis inhibition pathway via LCN2 in normal human FTE and human FTE with p53 dysfunction. This diagram highlights the role of LCN2 in mediating ferroptosis resistance under both conditions. Created with BioRender.com. Data are represented as the mean ± SEM, in principle. Statistical analyses were performed using the paired t-test for Figure 7B and nonlinear regression with the extra sum-of-squares F test for Figure 7H, while the Student’s *t* tests were used for comparisons between two groups in all other cases. Significance levels are ∗*p* < 0.05, ∗∗*p* < 0.01, ∗∗∗*p* < 0.001, and ∗∗∗∗*p* < 0.0001.

The human FT is not only a tunnel for an ovum but also a starting point for tumorigenesis of HGSC.^26^ Most HGSC cases possess *TP53* pathogenic mutations; therefore, certain changes triggered by p53 dysfunction may promote tumorigenesis in HGSC. Moreover, p53 suppresses NF-κB signaling.^27^ From these previous reports, it was hypothesized that tumorigenesis due to the p53 dysfunction is relevant to the stemness in the human FTE. First, we investigated the expression of LCN2 by immunofluorescence in a sample of very early-stage HGSC (Stage IA) diagnosed following risk-reducing salpingo-oophorectomy (RRSO) in a *BRCA2* pathogenic variant carrier. Compared to the normal FTE in the sample, very early-stage HGSC regions highly expressed LCN2 (Figure 7A), indicating that p53 dysfunction can fuel the expression of LCN2. We also examined the expression of LCN2 by IHC in the epithelial lesions with abnormal p53-staining, specifically serous tubal intra-epithelial carcinoma (STIC), which were identified in 18 clinical FTE samples. As a result, an increased expression of LCN2 was observed in the FTE lesions with abnormal p53-staining (Figure 7B).

To investigate the cellular changes in LCN2 expression and NF-κB signaling caused by p53 dysfunction in the human FTE, we established human FTE organoids with *TP53* knockout (p53-KO) by CRISPR/Cas9 and selected the p53-KO organoids by 10 μM Nutlin-3A treatment or single organoid cloning (Figures 7C and 7D). The genetic manipulation in the locus of *TP53* of the single-picked p53-KO organoids was confirmed by whole exome sequencing (Table S1). Next, we investigated the changes in differentiation status caused by p53 dysfunction using qPCR. As a result, p53 dysfunction reduced the expression of the differentiation marker genes *OVGP1* and *FOXJ1* (Figure 7E). We also examined the expression of *LCN2* in the p53-KO organoids by qPCR, and found that the expression of *LCN2* was fueled by p53 dysfunction (Figure 7F). The activation of NF-κB signaling in the p53-KO organoids was then examined. Compared to the human FTE organoids, the expression of NF-κB signaling downstream genes (*TNF*, *IL1A*, *IL1B*, and *IL6*) was increased by 4.23-, 6.40-, 4.87-, and 2.75-fold, respectively (Figure 7G). These results indicated that p53 dysfunction increases *LCN2* expression and activates NF-κB signaling in the human FTE.

A cell growth assay showed that the p53 dysfunction did not change the growth rate of the human FTE organoids (Figure 7H). Finally, to investigate whether the p53 dysfunction affects resistance to ferroptosis, we performed the sensitivity assays on the human FTE organoids and the p53-KO organoids using artemisinin. Consequently, p53 dysfunction enhanced ferroptosis resistance by 1.29-fold (Figure 7I).

## Discussion

Understanding the histological and anatomical structures that constitute human organs and their homeostasis is crucial for understanding their functions and roles in disease development. The organoid culture method recapitulating human tissues *in vitro* is well-suited for analyzing the heterogeneity of cell functions and properties within tissues.^19^ In this study, we established human FTE organoids and analyzed the heterogeneity of cellular proliferation using the fluorescent dye, PKH26. This revealed that the proliferation rates of individual cells within the early-culture organoid varied, and quiescent cells and proliferative cells were present in the undifferentiated FTE organoids. Upon analyzing the characteristics of quiescent cells using bRNA-seq analysis, we observed the downregulation of signaling related to cell proliferation. Additionally, it was found that the quiescent cells exhibited transcriptomic characteristics of quiescent stem cells, such as downregulated mTORC1 signaling and oxidative phosphorylation, reported in other tissues.^14^^,^^20^ The organoid formation assay (Figure 2F) also showed that both the PKH26-retained cells and PKH26-reduced cells could develop human FTE organoids, and the organoid expansion rate of the PKH26-reduced cells was higher than that of the PKH26-retained cells. These results suggested that the pre-matured human FTE organoids cultured for around one week comprised the PKH26-retained, quiescent stem-like cells and the PKH26-reduced, proliferative progenitor-like cells. Long-term organoid culture over two weeks gradually led to the increased expression of late-stage differentiation marker genes such as *OVGP1* and *FOXJ1*. Additionally, the folded structures and α-tubulin-positive cilia emerged, which indicated that the individual cells in the pre-matured FTE organoids differentiated during long-term organoid formation leading the organoids to recapitulate the human FTE. The quiescent cells which retained PKH26 observed in the pre-matured organoids validated the use of early-culture organoids for the identification and characterization of quiescent stem-like cells in the human FTE. Furthermore, we discovered that NF-κB signaling, which canonically functions as an inflammatory mediator, was activated in the quiescent cells within the human FTE organoids, and also in the quiescent cell regions of the human FT scRNA-seq data. NF-κB signaling is known for its representative role in inflammatory responses, its contribution to quiescence and survival control of hematopoietic stem cells, and the maintenance of neural stem cells under stress conditions, indicating its close association with stemness in other organs.^16^^,^^28^ However, there are only a few reports on its relevance to stem cell properties in epithelial tissues. In this study, although the function of NF-κB signaling in the human FTE stem cells remains unclear, it is intriguing that the association has at least been suggested.

Tissue stemness is based on a complex network of genes and molecules, making it difficult to capture it comprehensively through a single mechanism or marker. Therefore, we assessed stemness using the dormancy gene set including 47 genes (Figure 3G). Additionally, by analyzing the two independent scRNA-seq data, we aimed to minimize biases from research methods and patient backgrounds. As a result, we successfully identified *LCN2* encoding a secreted protein that can contribute to niche development and is suggested to be associated with NF-κB signaling, as a gene involved in the stemness in the human FTE (Figure 7J). LCN2 is a secreted protein known to possess an antibacterial activity by binding to bacterial siderophores, thereby inhibiting the uptake of ferric ions.^22^ In addition to its role in fighting infection, LCN2 is involved in various physiological processes, such as inflammation, immune response, and iron metabolism. It has been found to play a particularly important role in suppressing ferroptosis, a type of programmed cell death distinct from apoptosis and characterized by the accumulation of lipid peroxides and the iron-dependent oxidative damage mediated by ferric ions.^23^ However, the role of LCN2 in epithelial cells remains unclear.

The human FTE microenvironment is subject to various changes; for instance, the human FTE is exposed to follicular fluids comprising various factors, hormones, and ions, approximately once in a month.^29^^,^^30^ As confirmed by our *in vitro* validation, ferric ions induce cell death in the human FTE cells in a concentration-dependent manner. In contrast, the overexpression of *LCN2* in the human FTE cells conferred resistance to ferroptosis, as demonstrated by the cell viability assays. These results suggested that in human FTE cells, LCN2 enables cell survival under oxidative stress conditions, such as exposure to cytotoxic ferric ions. Given that stem cells in the tissue express high levels of *LCN2*, and the high expression decreases upon differentiation, it can be inferred that the LCN2-mediated protective mechanism against oxidative stress is closely related to the establishment of a stem cell niche (Figure 7J). In other organs, the resistance of tissue stem cells to reactive oxygen species has been confirmed through various mechanisms,^31^ indicating its importance in maintaining homeostasis. Therefore, the discovery of oxidative stress resistance mediated by LCN2 in this study is significant, and advances our understanding of the mechanisms that maintain tissue homeostasis in the human FTE.

From the perspective of gynecological diseases, it is well known that the human FTE is the site of origin of HGSC, one of the most lethal gynecological malignancies.^26^ HGSC is characterized by pathogenic mutations in the tumor suppressor gene *TP53* in most cases, and the p53 dysfunction has been suggested to play a significant role in the carcinogenesis of HGSC. Generally, cancer cells exhibit abnormally increased cell proliferation.^32^ Intriguingly, in human FTE organoids, p53 dysfunction did not promote cell proliferation. This suggested that p53 dysfunction induces carcinogenesis in the human FTE cells through mechanisms other than cell proliferation. Elucidating these mechanisms is expected to enhance our understanding of the carcinogenesis of HGSC.

p53 is known to be relevant to NF-κB signaling regulation.^27^ The qPCR results showed that the expression of NF-κB downstream genes (*TNF*, *IL1A*, *IL1B*, and *IL6*) was enhanced by the p53 dysfunction; furthermore, the expression of *LCN2* was also upregulated by the p53 dysfunction. It is also known that the p53 dysfunction in the FT suppresses differentiation into ciliated cells,^33^ and the decrease in *FOXJ1* expression by the p53 dysfunction was confirmed by the qPCR result. These findings indicate that the p53 dysfunction induces the changes in the cell population of the human FTE, resulting in the loss of the original gradation in differentiation status established by the distribution of *OVGP1* (+) late-stage secretory cells and *FOXJ1* (+) ciliated cells, which are downstream of epithelial cell differentiation and an increase in undifferentiated cells. Such changes in cell population indicate a tendency toward the loss of heterogeneity in the human FTE. Furthermore, the upregulation of *LCN2* expression is thought to enhance the resistance to ferroptosis and oxidative stress in the human FTE, creating a favorable environment for the growth of cancer or precancerous cells by disrupting the balance between cell death and survival through the defense mechanism. Thus, we propose that p53 dysfunction in human FTE cells, through the increase in NF-κB signaling-activated cells, the rise in undifferentiated cells, and the upregulation of *LCN2* expression, disrupts the equilibrium between the stemness and differentiation heterogeneity, thereby promoting carcinogenesis and progression (Figure 7J). Indeed, in the analysis of occult cancer tissue identified during RRSO which is considered to be the earliest HGSC, LCN2 also showed corresponding localization with the aberrant p53 staining, indicating that the increased expression of *LCN2* is associated with the early stages of HGSC carcinogenesis.

While previous studies have focused on specific pathways or molecules to investigate stem cells in the human FT, our research provides a new perspective by focusing on the heterogeneity of cell proliferation and integrating organoid culture technology with open science to explore the stemness of the human FTE.

In conclusion, this study is significant as it reveals that LCN2 is involved in the stemness of the human FTE, enhancing our understanding of tissue homeostasis and mechanisms of HGSC development. Furthermore, the high expression of LCN2 that can be measured via blood tests in the very early stages of HGSC development suggests that LCN2 may serve as an early diagnostic marker for HGSC. This research provides a new perspective on the stemness of the human FTE and early cancer detection, and is anticipated to be a solid foundation for future research and clinical applications.

### Limitations of the study

Although we utilized organoid culture technology, we did not replicate the interactions with other cells, such as stromal or immune cells. In addition, the number of clinical samples used was limited. To overcome these limitations, it is necessary to introduce new systems, such as co-culture with different cell types, and conduct further tissue analyses. In addition, while the dormancy gene set and the marker gene *LCN2* identified in this study may reflect characteristics of FTE stem cells, we did not perform *in vitro* assays to confirm stem cell properties such as multipotency or long-term self-renewal.

## Resource availability

### Lead contact

Requests for further information and resources should be directed to and will be fulfilled by the lead contact, Kenta Masuda (ma-su-ken.a2@keio.jp).

### Materials availability

Materials generated in this study are available upon reasonable request and require a material transfer agreement.

### Data and code availability

The bRNA-seq data have been deposited in the GEO with an accession number GSE274309. The source codes for reproducing the results in this paper and the environment settings were deposited in the GitHub page of Tomohiro Tamura: “https://github.com/Tomo-koko/Human-fallopian-tube-stemness-project”.

## Acknowledgments

The authors thank the Core Facility, Collaborative Research Resources, and the JSR-Keio University Medical and Chemical Innovation Center (JKiC) for their help with the experiments. The authors thank A. Aoki, H. Nakazawa, and I. Ishimatsu for their assistance. The authors also appreciate the technical assistance and useful advice from the members of Department of Extended Intelligence for Medicine, the Ishii-Ishibashi Laboratory, Keio University School of Medicine. This research was supported by 10.13039/100009619AMED under grant number: JP23ama221509, 10.13039/501100000646JSPS KAKENHI grant numbers: JP20K18174, JP23K06640, and JP23KK0159, and JSPS Research Fellowship grant number: 24KJ1940, and Moonshot R&D - MILLENNIA Program (JPMJMS2022-19 to O.N.) of the Japan Science and Technology Agency (JST).

## Author contributions

Conceptualization: K.I., Tomohiro Tamura, and K.M.; methodology: K.I., Tomohiro Tamura, S.N., E.S., J.Y., Kohei Nakamura, Takashi Takeda, R.Y., and K.M.; software: Tomohiro Tamura; investigation: K.I. and Tomohiro Tamura; formal analysis: K.I., Tomohiro Tomura, and Kohei Nakamura; data curation: K.I. and Tomohiro Tamura; writing–original draft: K.I. and Tomohiro Tamura; writing–review and editing: Takashi Takeda, Kanako Nakamura, Y.N., K.T., I.K., T.C., Y.K., K.B., K.S., H.S., D.A., A.A.A., O.N., K.M., and W.Y.; visualization: K.I. and Tomohiro Tamura; supervision: K.M. and W.Y.; resources: Y.O., O.N., K.M., and W.Y.; project administration: K.M.; funding acquisition: K.M.

## Declaration of interests

The authors declare no conflicts of interest associated with this manuscript.

## STAR★Methods

### Key resources table

**Table:** 

REAGENT or RESOURCE	SOURCE	IDENTIFIER
**Antibodies**		

anti-α-tubulin (6-11B-1)	Santa-cruz	sc-23950 RRID:AB_628409
anti-PAX8 (1F8-3A8)	Invitrogen	MA1-117 RRID:AB_2536828
anti-human LCN2	Bio-techne	AF1757 RRID:AB_354974
anti-p53	Cell signaling	2527 RRID:AB_10695803

**Bacterial and virus strains**		

NEB Stable Competent *E. coli* (High Efficiency)	New England Biolabs	C3040

**Biological samples**		

Human fallopian tube samples	Keio University Hospital	N/A
Human serous tubal intraepithelial carcinoma samples	Keio University Hospital	N/A
Human ovarian cancer samples	Keio University Hospital	N/A

**Chemicals, peptides, and recombinant proteins**		

Advanced DMEM/F12	Thermo Fisher Scientific	12634010
HEPES	gibco	15630–106
GlutaMAX-I	gibco	35050–061
R-spondin CM	JSR Life Science or In-house	N/A
Noggin CM	JSR Life Science or In-house	N/A
B27	gibco	17504–044
N-acetyl-L-cysteine	Sigma-Aldrich	A9165
A83-01	Tocris	2939/10
human EGF	Peprotech	AF-100-15
human FGF-10	Peprotech	100–26
*N*-2 supplement	gibco	17502–048
Forskolin	Wako	067–02191
Nicotinamide	Sigma-Aldrich	N0636
Y27632	Sigma-Aldrich	Y0503
Matrigel Matrix	Corning	354230
Artemisinin	Cayman	11816
Erastin	Cayman	17754
PKH26	Sigma-Aldrich	MINI26

**Critical commercial assays**		

PrimeScript RT Reagent Kit with gDNA Eraser (Perfect Real Time)	Takara	RR047
CellTiter-Glo 2.0	Promega	G9243
CellTiter-Glo 3D	Promega	G9681
Illumina TruSeq Stranded mRNA Library Prep Kit	Illumina	RS-122-2101

**Deposited data**		

RNA sequencing data -PKH26 sorting	This paper	GEO: GSE274309
Single cell RNA sequencing data by Lengyel et al.	Lengyel et al., *Cell Reports*^4^ CELLxGENE	https://datasets.cellxgene.cziscience.com/9e7dd889-c863-46f9-9ab9-99175e23fd75.rds
Single cell RNA sequencing data by Ulrich et al.	Ulrich et al., *Developmental cell*^5^ CELLxGENE	https://datasets.cellxgene.cziscience.com/116cae82-6420-48a8-af2f-bc9f64545788.rds

**Oligonucleotides**		

Human *ACTB* forward: AGGCACCAGGGCGTGAT	N/A	N/A
Human *ACTB* reverse: GCCCACATAGGAATCCTTCTGAC	N/A	N/A
Human *MKI67* forward: AAGCCCTCCAGCTCCTAGTC	N/A	N/A
Human *MKI67* reverse: TCCGAAGCACCACTTCTTCT	N/A	N/A
Human *OVGP1* forward: ATCGGCGGGTGGAACTTTG	N/A	N/A
Human *OVGP1* reverse: TGGGGCTGCCTCTTAGTCC	N/A	N/A
Human *FOXJ1* forward: CACGGACAACTTCTGCTACTT	N/A	N/A
Human *FOXJ1* reverse: GGCACTTTGATGAAGCACTTG	N/A	N/A
Human *LCN2* forward: AGCAGAACTTCCAGGACAAC	N/A	N/A
Human *LCN2* reverse: TTGCGGGTCTTTGTCTTCTC	N/A	N/A
Human *TP53* forward: GTACCACCATCCACTACAACTAC	N/A	N/A
Human *TP53* reverse: CACAAACACGCACCTCAAAG	N/A	N/A
Human *IL4I1* forward: GGCACACGCTCTTGGAATA	N/A	N/A
Human *IL4I1* reverse: GGCGAAGCTGAGATAGAAGAAG	N/A	N/A
Human *HLA-DPA1* forward: GGCACCGTCCTCATCATAAA	N/A	N/A
Human *HLA-DPA1* reverse: ATCTCTCCTAAGTCCTCTTCTGT	N/A	N/A
Human *HLA-DPB1* forward: ATTCTGCCCGGAGTAAGACAT	N/A	N/A
Human *HLA-DPB1* reverse: TCGTTGAACTTTCTTGCTCCTC	N/A	N/A
Human *HLA-DQA1* forward: TGGGCACTGTCTTCATCATC	N/A	N/A
Human *HLA-DQA1* reverse: GTCCATTCTTCTGCTCCTGTAG	N/A	N/A
Human *HLA-DQB1* forward: ATCCGTCAAAGGAGTCAGAAAG	N/A	N/A
Human *HLA-DQB1* reverse: AAGCAGGCATCACAGAAGAG	N/A	N/A
Human *HLA-DRA* forward: GGTCTGGTGGGCATCATTATT	N/A	N/A
Human *HLA-DRA* reverse: CATCACCTCCATGTGCCTTAC	N/A	N/A
Human *HLA-DRB1* forward: ACAGAGCAAGATGCTGAGTG	N/A	N/A
Human *HLA-DRB1* reverse: CTGAAGTCCAGAGTGTCCTTTC	N/A	N/A
Human *TNF* forward: CCAGGGACCTCTCTCTAATCA	N/A	N/A
Human *TNF* reverse: TCAGCTTGAGGGTTTGCTAC	N/A	N/A
Human *IL1A* forward: TGTGACTGCCCAAGATGAAG	N/A	N/A
Human *IL1A* reverse: CGTGAGTTTCCCAGAAGAAGAG	N/A	N/A
Human *IL1B* forward: ATGGACAAGCTGAGGAAGATG	N/A	N/A
Human *IL1B* reverse: CCCATGTGTCGAAGAAGATAGG	N/A	N/A
Human *IL6* forward: CACTCACCTCTTCAGAACGAAT	N/A	N/A
Human *IL6* reverse: GCTGCTTTCACACATGTTACTC	N/A	N/A

**Recombinant DNA**		

pEF1A-hLCN2 plasmid	VectorBuilder	VB900000-0557pkp

**Software and algorithms**		

inForm	PerkinElmer	N/A
Incucyte Organoid Analysis Software	Sartorius	N/A
StrataQuest version 7	TissueGnostics	N/A
Prism	GraphPad	N/A
R	N/A	N/A

**Other**		

Source code of the analysis in this paper	GitHub page of the author To.T.	https://github.com/Tomo-koko/Human-fallopian-tube-stemness-project

### Experimental model and study participant details

A normal FTE tissue sample from a 48-year-old participant with benign gynecologic disease and samples of FTE lesions with abnormal p53-staining from 18 participants with the average age: 57.78 ± 2.089 (mean ± SEM) including an early-stage HGSC sample from a participant with germline *BRCA2* pathogenic variant were collected from the University Hospital with the approval of the Institutional Ethics Committee (Approval No. 20070081, 20210111). All participants in this study were Asian women. All procedures involving human participants were performed in accordance with the ethical standards of the institutional and/or national research committee or both. This study conformed to the Declaration of Helsinki (1964) and its later amendments or comparable ethical standards. Written informed consent was obtained from each participant. The tissue samples were fixed in formalin and paraffin-embedded (FFPE).

### Method details

#### Tissue processing, organoid culture of FT samples, and viral gene engineering

For establishing normal FTE organoids, the FT fimbriae were dissected, and digested with collagenase type I at 37°C, followed by incubation in TrypLE at 37°C. Dispersed FTE cells were mixed with Matrigel (Corning), and 20–25 μL drops of matrix cell suspension were allowed to solidify on a 48-well plate at 37°C. To stabilize the Matrigel, an organoid medium was added. The organoid medium was based on Advanced DMEM/F12 (Thermo Fisher Scientific) supplemented with 10 mM HEPES (gibco), 2 mM GlutaMAX-I (gibco), 50 × B27 (gibco), 1 mM N-acetyl-L-cysteine (Sigma-Aldrich), R-spondin CM (JSR Life Science or In-house), Noggin CM (JSR Life Science or In-house), 100 ng/mL human EGF (Peprotech), 100 ng/mL human FGF-10 (Peprotech), 100 × *N*-2 supplement (gibco), 1 mM Nicotinamide (Sigma-Aldrich), 10 μM Forskolin (FUJIFILM), and 5 μM A83-01 (Tocris), in principle. To add the first organoid medium at establishment or passage, the culture medium was supplemented with 10 μM Y-27632 (Sigma-Aldrich). Normal FTE organoids were transfected with the pEF1A-hLCN2 plasmid (VectorBuilder, VB900000-0557pkp) (LCN2-OE). After transfection, the medium was replaced with a medium containing 5 μg/mL puromycin (InvivoGen).

#### CRISPR/Cas9-mediated knockout of *TP53*

Organoids derived from early passaged (P0-P3) FTE organoids were trypsinized and the cells were collected in electroporation cuvettes (NepaGene) with Opti-MEM (Thermo Fisher Scientific), an appropriate sgRNA, Cas9 Nuclease, and Cas9 Electroporation Enhancer. Electroporation was performed using NEPA21 (NepaGene). After electroporation, the cells were incubated for 30 min, mixed with Matrigel, and covered with the organoid medium containing 10 μM Y-27632. Approximately 2 days post-transfection, the medium was exchanged with a medium containing 10 μM Nutlin-3a to select *TP53*-knocked organoids. sg*TP53* RNA was synthesized using IDT Alt-R CRISPR-Cas9 sgRNA *TP53* TCCACTCGGATAAGATGCTG.

#### Cell proliferation assay

Human FTE organoids were cultured in 48-well plates. ATP levels were measured using CellTiter-Glo 3D (Promega), according to the manufacturer’s instructions, and luminescence was measured using a Cytation5 plate reader (BioTek).

#### Cell sorting by proliferation using PKH26

Human FTE organoids were dissociated into single cells using Cell Recovery Solution and TrypLE. The cells were labeled with PKH26 (Sigma-Aldrich), according to the manufacturer’s instructions. Briefly, the cells were suspended in Diluent C and incubated with PKH26 dye for 3 min at room temperature. The reaction was terminated using FBS and the cells were washed with Advanced DMEM. The labeled cells were resuspended in PBS and sorted into two populations using a MoFlo XDP cell sorter (Beckman Coulter) on day6. The two populations were as follows: PKH26-retained cells, representing approximately the top quarter of the stained cells based on fluorescence intensity, and PKH26-reduced cells, whose fluorescence intensity was similar to that of the negative control.

#### Quantitative-PCR (qPCR)

Total RNA was isolated from the organoids using a RNeasy Mini Kit (Qiagen), followed by the removal of genomic DNA and reverse transcription using a PrimeScript RT Reagent Kit with gDNA Eraser (Perfect Real Time) (Takara). Reverse transcription (RT)-qPCR was performed using the CFX Duet Real-Time PCR System (Bio-Rad).

#### Immunohistochemistry

Human FTE organoids were collected from culture plates, fixed in formalin, and embedded in paraffin. FFPE slides, including those prepared from organoids and clinical specimens, were deparaffinized using Clear Plus and ethanol, followed by antigen retrieval. Endogenous peroxidase activity was blocked, and the slides were incubated with primary antibodies diluted in TBS or TBS-T for 1 or 2 h at room temperature. After washing, secondary antibodies diluted in TBS or TBS-T were applied for 30 min at room temperature. Signal detection was performed using the ABC Reagent Vectastain kit and DAB substrate. Finally, the slides were counterstained with hematoxylin and eosin (H&E) and mounted. Antibody details are provided in the key resource table. The protocol for immunohistochemistry of STIC in Figure 7B was optimized individually.

#### Quantification of LCN2 expression within human FTE by IHC

The samples stained with the anti-LCN2 antibody from 18 clinical samples of human FTE containing epithelial lesions with abnormal p53-staining were scanned using a NanoZoomer digital slide scanner (Hamamatsu). The resulting images were analyzed using StrataQuest version 7 software (TissueGnostics). Both of the normal epithelium and the epithelial lesions with abnormal p53-staining were identified separately, and the total epithelial area was defined for the analysis of each area. The LCN2-positive area was quantified as a fraction of the total epithelial area.

#### Multiplex immunofluorescence

The FFPE specimen slides were de-paraffinized using Clear Plus and ethanol. Antigen retrieval was performed using the Target Retrieval Solution (Dako) at 120°C. The slides were placed in TBS-T after quenching. Specimens were permeabilized with 3% H_2_O_2_/TBS and blocked with 3% BSA/TBS at room temperature. The membranes were incubated with primary antibodies (key resource table) for 1 h at room temperature. After washing, the membranes were incubated with secondary antibodies for 1 h at room temperature. The specimens were counterstained with Hoechst33342 and mounted using ProLong Diamond Antifade Mountant (Thermo Fisher, P36961). Slides were scanned with a Vectra Polaris, and the fluorescent images were processed using the software "inForm”.

#### Drug sensitivity assay

The cells harvested using TrypLE were counted with a cell counter using trypan blue. Cells were seeded in 96-well plates on day 1. The indicated concentrations of Artemisinin, Erastin, or vehicle were added when the culture medium was refreshed on day 4. ATP levels were measured using CellTiter-Glo 2.0 (Promega), according to the manufacturer’s instructions, and luminescence was measured on day 6 using a Cytation5 plate reader (BioTek). The results were normalized to those of the control samples and analyzed.

#### Comparison of organoid forming efficiency

Cells (2,000/well) were embedded in 20 μL of Matrigel and seeded into a 48-well plate. Organoid cultures were imaged over 10 days using the Incucyte 3S Live-Cell Analysis System (Sartorius), housed in an incubator. Bright-field images were captured every 6 h at 4× magnification. The counts and total area of organoids were analyzed using the Incucyte Organoid Analysis Software, according to the manufacturer’s instructions, with organoids defined as structures with an area of ≥10,000 μm^2^. Wells exhibiting air bubbles or Matrigel debris at the time of seeding were excluded from the analysis to ensure accuracy.

#### Whole-exome sequencing

Genomic DNA was extracted from the p53-KO organoids using the QIAamp DNA Mini Kit (Qiagen). DNA quality was evaluated using an Agilent 4200 TapeStation, and only samples with a DIN ≥2.0 were used. Sequencing libraries were prepared with the SureSelectXT Low Input Target Enrichment System (Agilent Technologies) using a custom panel targeting 216 genes based on RefSeq annotations. Sequencing was performed on a NextSeq 2000 system (Illumina) with 150 bp paired-end reads. Sequence data were processed with the GenomeJack pipeline (Mitsubishi Electric Software Corporation), and samples with mean depth <200 or read remain rate <70% were reanalyzed. Copy number variation (CNV) was calculated from normalized read depths and categorized as amplified, amplified-like, loss-like, or deleted based on log2 ratios and variability metrics.

#### Bulk RNA sequencing (bRNA-seq) analysis

Cultured organoids were subjected to RNA extraction using a RNeasy Plus Mini Kit (Qiagen), according to the manufacturer’s instructions. RNA quality was assessed using an Agilent 2100 Bioanalyzer, ensuring a RIN value >7 for all samples. RNA libraries were prepared using a TruSeq Stranded mRNA Library Prep Kit (Illumina). Multiplexed library pools were sequenced using 151 bp paired-end reads on the NovaSeq6000 platform (Illumina). Quality control of the raw sequencing reads was performed using the FastQC software. Clean reads were aligned to the human reference genome (GRCh38) using HISAT2. TPM values of each gene in the two samples produced, according to the abovementioned process, were deposited into the Gene Expression Omnibus (GEO) database.

For GSEA, we utilized the R package “fgsea”.^34^ We created the preranked gene vector by the TPM ratio of the PKH26-retained cells to the PKH26-reduced cells. To avoid the division by zero, we added a very small number (0.01) to all the TPM values in advance. The gene sets included in Molecular Signatures Database (MSigDB) were obtained by the R package “msigdbr”.^35^^,^^36^ To examine the enrichment of a cell cycle-related gene set, we drew the bar cord plot of the gene set “GOBP_CELL_CYCLE” (Human:C5:BP). To explore upregulated or downregulated gene sets in the PKH26-retained cells, we performed GSEA in terms of human hallmark gene sets (Human:H).

To establish the dormancy gene set including the genes “upregulated in the PKH26-retained cells compared with the PKH26-reduced cells” and “highly-expressed in the PKH26-reduced cells”, we searched for the genes meeting the formula: log_10_(normalized TPM ratio of the PKH26-retained cells to the PKH26-reduced cells) × log_10_(TPM value of the PKH26-retained cells) > 2. Finally, we converted the gene symbols to the corresponding Ensembl IDs by the R package “biomaRt” (“ENSEMBL_MART_ENSEMBL”, dataset: hsapiens_gene_ensembl, version: 110) for single-cell RNA sequencing (scRNA-seq) analysis described later.^37^

#### scRNA-seq analysis

Firstly, we imported the human fallopian tube scRNA-seq data RDS files from the CZ CELLxGENE sites; Lengyel et al.: “https://datasets.cellxgene.cziscience.com/9e7dd889-c863-46f9-9ab9-99175e23fd75.rds”, Ulrich et al.: “https://datasets.cellxgene.cziscience.com/116cae82-6420-48a8-af2f-bc9f64545788.rds”. The data by Lengyel et al. included 60,574 cells, and that by Ulrich et al. included 59,738 cells. To investigate epithelial cells in the scRNA-seq data, we examined the expression of *EpCAM* (Ensembl ID: ENSG00000119888) and *CDH1* (Ensembl ID: ENSG00000039068) of all the cells, and extracted *EpCAM*/*CDH1* (+/+) epithelial cells by the formula: the normalized expression of *EpCAM* (Ensembl ID: ENSG00000119888) > 0.1 and the normalized expression of *CDH1* (Ensembl ID: ENSG00000039068) > 0.1 (Lengyel et al.: 2,791 cells, Ulrich et al.: 9,678 cells). To analyze the single-cell-level enrichment of the dormancy gene set defined by the bRNA-seq results, we calculated the enrichment score by the R package “escape” and its dependency “AUCell”, and attached the score as metadata to the scRNA-seq data.^38^^,^^39^

Next, to examine the activation of major pathways, especially NF-κB signaling, we utilized the R package “progeny”.^40^ In advance, we prepared PROGENy objects which included log-scale expression values and added a very small number (0.01) to avoid “log_10_0” from the two scRNA-seq data. All Ensembl IDs were converted to HGNC Hugo Symbols except for Ensembl IDs assigned no gene symbol. When Ensembl IDs represented two genes, the updated Ensembl database was referred and one gene was selected for one Ensembl ID. When genes possessed two or more Ensembl IDs, we adopted the most highly expressed Ensembl IDs, or selected one Ensembl ID randomly from top-tier Ensembl IDs. Based on the preparation, we built the PROGENy objects in which Ensembl IDs represented gene one-to-one. Subsequently, the activation score of major pathways was calculated, and the activation scores were attached as metadata to the scRNA-seq data.

To perform a trajectory analysis in addition to the basic scRNA-seq analysis, we utilized the R package “monocle3” and its dependency “batchelor”.^41^^,^^42^^,^^43^^,^^44^^,^^45^ We created CDS objects from the two scRNA-seq count and metadata. PCA was conducted and the batch effects caused by donor IDs were removed, followed by UMAP, clustering, and learning trajectory graphs (without pseudotime), and the expression of conventional differentiation marker genes in human FTE were examined: *PAX8* (Ensembl ID: ENSG00000125618), *KRT7* (Ensembl ID: ENSG00000135480), *OVGP1* (Ensembl ID: ENSG00000085465), *FOXJ1* (Ensembl IDs: ENSG00000129654), and *CAPS* (Ensembl ID: ENSG00000105519). To calculate the pseudotime in the differentiation of human FTE, we identified the root node which was the nearest node to the stem-like cells with the highest enrichment score of the dormancy gene set, and calculated the pseudotime from the root node. Searching for genes whose expressions transited along the pseudotime by Moran’s I test, found 1,288 Ensemble IDs were obtained as the intersection of 1,363 Ensembl IDs from the data by Lengyel et al. and 4,157 Ensembl IDs from the data by Ulrich et al. by the formula: q-value = 0.

Finally, to detect the important genes in the stemness of human FTE, we searched for genes whose expressions decreased monotonically along the pseudotime. To analyze the transition of each gene’s expression, we modeled the transition with natural spline functions and calculated the derivatives at all the defined pseudotime by the R package “splines2”.^46^ We extracted 14 Ensembl IDs with the formula: the derivatives at all the defined pseudotime <0 in both of the two scRNA-seq data.

Please refer to the “Data and code availability” section in terms of the parameters in each calculation and modeling, and the computational environment settings.

### Quantification and statistical analysis

GraphPad Prism software was used for statistical analyses, except for the bRNA-seq analysis and the scRNA-seq analysis. For comparisons between two groups, the Student’s t test was used basically. For a comparison between two groups related to each other, the paired t-test was applied. For analyses involving linear and nonlinear regression models, the extra sum-of-squares F test was applied, as required for each figure. The values are expressed as the means ± standard error of the mean (SEM), in principle. Significance levels are ∗*p* < 0.05, ∗∗*p* < 0.01, ∗∗∗*p* < 0.001, and ∗∗∗∗*p* < 0.0001.
