# Supplementary material for: LCN2-mediated ferroptosis resistance in tissue homeostasis and early-stage tumorigenesis of the fallopian tube epithelium
Source: iScience. 2025 May 13;28(6):112654. doi: 10.1016/j.isci.2025.112654 (PMC12158494; doi:10.1016/j.isci.2025.112654)
Supplement: Document S1. Table S1 [file mmc1.pdf]

iScience, Volume *28*

Supplemental information

LCN2-mediated ferroptosis resistance in tissue homeostasis and early-stage tumorigenesis of the fallopian tube epithelium

Keiyo Imaeda, Tomohiro Tamura, Shimpei Nagai, Eiji Sugihara, Juntaro Yamasaki, Yuji Otsuki, Kohei Nakamura, Takashi Takeda, Kanako Nakamura, Yuya Nogami, Kosuke Tsuji, Tatsuyuki Chiyoda, Iori Kisu, Yusuke Kobayashi, Kouji Banno, Rui Yamaguchi, Kazuhiro Sakurada, Hideyuki Saya, Daisuke Aoki, Ahmed Ashour Ahmed, Osamu Nagano, Kenta Masuda, and Wataru Yamagami

	Gene	AA Syntax	CDS Syntax	VAF	SnpEff Effect	Chromosome
clone9	*TP53*	p.Q192Hfs*30	c.576_919+12delGCATCTTATCCGAGTGGAAGGAAATTTGCGTGTGGAGTATTTGGATGACAGAAACACTTTTCGACATAGTGTGGTGGTGCCCTATGAGCCGCCTGAGGTCTGGTTTGCAACTGGGGTCTCTGGGAGGAGGGGTTAAGGGTGGTTGTCAGTGGCCCTCCAGGTGAGCAGTAGGGGGGCTTTCTCCTGCTGCTTATTTGACCTCCCTATAACCCCATGAGATGTGCAAAGTAAATGGGTTTAACTATTGCACAGTTGAAAAAACTGAAGCTTACAGAGGCTAAGGGCCTCCCCTGCTTGGCTGGGCGCAGTGGCTCATGCCTGTAATCCCAGCACTTTGGGAGGCCAAGGCAGGCGGATCACGAGGTTGGGAGATCGAGACCATCCTGGCTAACGGTGAAACCCCGTCTCTACTGAAAAATACAAAAAAAAATTAGCCGGGCGTGGTGCTGGGCACCTGTAGTCCCAGCTACTCGGGAGGCTGAGGAAGGAGAATGGCGTGAACCTGGGCGGTGGAGCTTGCAGTGAGCTGAGATCACGCCACTGCACTCCAGCCTGGGCGACAGAGCGAGATTCCATCTCAAAAAAAAAAAAAAAAGGCCTCCCCTGCTTGCCACAGGTCTCCCCAAGGCGCACTGGCCTCATCTTGGGCCTGTGTTATCTCCTAGGTTGGCTCTGACTGTACCACCATCCACTACAACTACATGTGTAACAGTTCCTGCATGGGCGGCATGAACCGGAGGCCCATCCTCACCATCATCACACTGGAAGACTCCAGGTCAGGAGCCACTTGCCACCCTGCACACTGGCCTGCTGTGCCCCAGCCTCTGCTTGCCTCTGACCCCTGGGCCCACCTCTTACCGATTTCTTCCATACTACTACCCATCCACCTCTCATCACATCCCCGGCGGGGAATCTCCTTACTGCTCCCACTCAGTTTTCTTTTCTCTGGCTTTGGGACCTCTTAACCTGTGGCTTCTCCTCCACCTACCTGGAGCTGGAGCTTAGGCTCCAGAAAGGACAAGGGTGGTTGGGAGTAGATGGAGCCTGGTTTTTTAAATGGGACAGGTAGGACCTGATTTCCTTACTGCCTCTTGCTTCTCTTTTCCTATCCTGAGTAGTGGTAATCTACTGGGACGGAACAGCTTTGAGGTGCGTGTTTGTGCCTGTCCTGGGAGAGACCGGCGCACAGAGGAAGAGAATCTCCGCAAGAAAGGGGAGCCTCACCACGAGCTGCCCCCAGGGAGCACTAAGCGAGGTAAGCAAGCAG	19.4%	HIGH	chr17
	TP53	p.V197Rfs*13	c.585_586insGGAG	16.1%	HIGH	chr17
	TP53	p.P190_I195del	c.567_584delCCCTCCTCAGCATCTTAT	40.9%	HIGH	chr17
	TP53	p.P191Sfs*52	c.571_583delCCTCAGCATCTTA	13.1%	HIGH	chr17
clone16	*TP53*	p.P191Kfs*9	c.570_595delTCCTCAGCATCTTATCCGAGTGGAAG	29.2%	HIGH	chr17
	TP53	p.H193Pfs*16	c.575_576insC	66.3%	HIGH	chr17

Supplemental Table 1: The genetic profiles of *TP53* locus analyzed by whole exome sequencing.
